# Whole-transcriptome sequencing reveals hypoxic esophageal squamous cell carcinoma–derived migrasomes driving cancer-associated fibroblast activation

**DOI:** 10.1093/bfgp/elag002

**Published:** 2026-06-02

**Authors:** Guan’en Qiao, Zhongping Wang, Meng Wang, Le Feng, Zixuan You, Bing Meng, Kui Dong, Yanping Cao, Pan Li, Junhai Wang, Xinqing Lu, Chunfang Xu

**Affiliations:** Department of Digestion, The First Affiliated Hospital of Soochow University, No. 188, Shizi Street, Gusu District, Suzhou 215006, Jiangsu Province, China; Department of Digestive, The First Hospital of Handan, No. 25 Congtai Road, Congtai District, Handan 056002, Hebei Province, China; Department of Geriatric, The First Hospital of Handan, No. 25 Congtai Road, Congtai District, Handan 056002, Hebei Province, China; Department of Digestive, The First Hospital of Handan, No. 25 Congtai Road, Congtai District, Handan 056002, Hebei Province, China; Department of Digestive, The First Hospital of Handan, No. 25 Congtai Road, Congtai District, Handan 056002, Hebei Province, China; Department of Digestive, The First Hospital of Handan, No. 25 Congtai Road, Congtai District, Handan 056002, Hebei Province, China; Department of Laboratory Medicine, The First Hospital of Handan, No. 25 Congtai Road, Congtai District, Handan 056002, Hebei Province, China; Department of Digestive, The First Hospital of Handan, No. 25 Congtai Road, Congtai District, Handan 056002, Hebei Province, China; Central Laboratory of the First Hospital of Handan, No. 25 Congtai Road, Congtai District, Handan 056002, Hebei Province, China; Department of Digestive, The First Hospital of Handan, No. 25 Congtai Road, Congtai District, Handan 056002, Hebei Province, China; Central Laboratory of the First Hospital of Handan, No. 25 Congtai Road, Congtai District, Handan 056002, Hebei Province, China; Department of Digestive, The First Hospital of Handan, No. 25 Congtai Road, Congtai District, Handan 056002, Hebei Province, China; Department of Digestion, The First Affiliated Hospital of Soochow University, No. 188, Shizi Street, Gusu District, Suzhou 215006, Jiangsu Province, China

**Keywords:** esophageal squamous cell carcinoma, hypoxia, migrasomes, CAF activation, whole-transcriptome sequencing

## Abstract

Cancer-associated fibroblasts (CAFs) activated by intercellular communication contribute to the progression of esophageal squamous cell carcinoma (ESCC). Migrasomes represent a novel mode of intercellular communication. However, the characteristics of ESCC-derived migrasomes in tumor hypoxic microenvironments and their effects on CAFs remain unclear. Migrasomes were isolated from ESCC cells under normoxia/hypoxia, with TSPAN4-GFP labeling, transmission electron microscopy, nanoparticle tracking analysis, and western blot for validation. Whole-transcriptome sequencing analyzed hypoxic migrasome RNA profiles, and ceRNA networks were predicted *via* RNAhybrid and Miranda. The effect of migrasomes on CAFs was assessed using fluorescence tracing, reverse transcription‑quantitative polymerase chain reaction, Transwell migration, enzyme‑linked immunosorbent assay, and western blot. ESCC cells produced migrasomes. Although hypoxia did not alter their quantity or structure, it significantly altered their RNA cargo, changing the composition of mRNA, lncRNA, and circRNA. Differentially expressed mRNAs were enriched in “Response to hypoxia” and “HIF-1 signaling pathway.” DElncRNAs were enriched in “Golgi to plasma membrane protein transport” and “Cell adhesion molecules pathway,” while DEcircRNAs were enriched in “ubiquitin binding” and “chromatin remodeling.” Predicted ceRNA networks were constructed using RNAhybrid and Miranda, involving 659 miRNAs, 24 lncRNAs, and 132 mRNAs. Fibroblasts internalized migrasomes and acquired a CAF-like phenotype, showing enhanced migration, elevated secretion of IL-1β/TGF-β, and increased CAF marker expression (α-SMA, COL1A1, COL3A1, FAP, PDGFRβ), with effects most pronounced under hypoxic migrasome treatment. This study characterized hypoxic migrasome whole transcriptome landscapes and suggested that hypoxic migrasomes may promote CAF-like changes *in vitro*, uncovering a novel ESCC–tumor microenvironment interaction mechanism and offering new perspectives for ESCC research.

## Introduction

Esophageal cancer represents the seventh most prevalent cancer worldwide [1]. Esophageal squamous cell carcinoma (ESCC), which initiates epithelial dysplasia, is a heterogeneous malignancy and accounts for over 85% of all esophageal cancers [2]. Morbidity and mortality of ESCC present an increasing trend year by year as the 5-year survival rate is as low as 20% [3, 4]. The treatment option for ESCC is limited compared to the other major types of cancer since ESCC represents a complex ecosystem in the tumor microenvironment (TME), which poses challenges for treatment [5]. This fact underscores the need for a deeper comprehension of novel mediums that regulate the TME in ESCC.

Cancer-associated fibroblasts (CAFs), an activated fibroblast subset, constitute the predominant stromal component within the TME [6]. Beyond their multifaceted roles in cancer progression [7], CAF regulation by tumor cells represents a core element of TME crosstalk. Illustratively, *Brca1*-deficient breast cancer cells induce *in vitro* transformation of CAFs into metastasis-associated fibroblasts with augmented proliferative, migratory, and invasive capacities [8]. Furthermore, hepatocellular carcinoma cell-derived exosomes carrying the RNPEP protein activate cancer-associated fibroblasts, thereby promoting tumor metastasis [9]. Stromal fibroblasts undergo glycolysis *via* tumor cell contact-initiated reprogramming, culminating in lactate production [10]. Nevertheless, the potential molecular factors underlying crosstalk between tumor cells and CAFs, particularly in ESCC, remain elusive.

Migrasomes are newly identified organelles forming on retraction fibers (RFs) of migrating cells. Postmigration, migrasomes break away from the retraction fibers, either undergoing spontaneous rupture or being engulfed by subsequent migratory cells, ultimately releasing the contents of donor cells [11]. Emerging evidence indicates that migrasomes exist in pancreatic cancer [12], gastric cancer [13], liver cancer [14], and glioblastoma [15], which play crucial roles in intercellular communication and significantly contribute to the remodeling of the TME [16]. Migrasomes originating from pancreatic cancer can induce macrophage polarization, which, in turn, fosters tumor growth [12]. Notably, Hu *et al.* revealed that Tetraspanin-4 (TSPAN4) + fibroblasts in pancreatic cancer exhibit upregulated migrasome-related gene expression and actively engage with immune cells *via* migrasomes, driving metastasis and immune evasion [17]. However, the regulatory effects of ESCC-derived migrasomes on CAFs remain unexplored.

Extracellular vesicles carry messengers that mediate intercellular communication, and among these messengers, noncoding RNAs (ncRNAs) emerging as key regulators of CAF functions in recent years. Studies demonstrate that tumor-derived exosomes deliver oncogenic ncRNAs such as LINC01915 in colorectal cancer, inducing transformation of normal fibroblasts into CAFs [18]. In breast cancer models, multiple exosomal ncRNAs—including lncRNA SNHG14 [19], miR-146a [20], and miR-370-3p [21]—have been shown to activate CAFs through distinct molecular mechanisms. Migrasomes constitute a distinct category of extracellular vesicles involved in intercellular communication. However, it remains unknown whether ESCC-derived migrasomes similarly activate CAFs *via* specific ncRNA cargo delivery.

To determine whether hypoxia-induced migrasomes from ESCC regulate CAF activation and functions *via* specific RNA cargo delivery, we employed integrated approaches including hypoxic cell models, migrasome isolation and characterization, and high-throughput whole-transcriptome sequencing. This systematic analysis elucidated the RNA profiles of ESCC migrasomes and their effects on CAFs. Our study not only reveals a novel migrasome-mediated pathway of tumor-stroma crosstalk but also identifies potential therapeutic targets for ESCC by disrupting migrasome-dependent TME interactions.

## Materials and methods

### Cell culture

The human ESCC cell line KYSE-150 (iCell, iCell-h245, China) was used in this study. The cell line was cultured in RPMI-1640 medium supplemented with 10% fetal bovine serum (FBS, 10099-141, Gibco, USA) and 1% penicillin–streptomycin (PS). Human embryonic lung fibroblasts MRC-5 cells (Procell, CL-0161, China) were cultured in MEM medium supplemented with 10% FBS and 1% PS. A hypoxic environment was established using hypoxia culture bags (1% O2 Genbag Microaer, 45532, bioMérieux, France). KYSE-150 was placed into the hypoxic culture bags and cultured under sealed conditions. All experiments were initiated from a standardized density of 1 × 10^7^ cells per condition for normalization.

Both cell lines were routinely authenticated by short tandem repeat (STR) profiling (KYSE-150: 2 November 2023; MRC-5: 10 November 2023). To ensure experimental consistency and minimize genetic drift, cells were resuscitated from low-passage-number cryopreserved stocks. Upon resuscitation and confirmation of robust growth, cells were expanded and aliquoted for cryopreservation to avoid repeated freeze–thaw cycles. Mycoplasma contamination was monitored monthly using a RT-qPCR-based detection method, and all cultures remained mycoplasma-negative throughout the study.

### Migrasome imaging in living cells

To label migrasomes derived from KYSE-150 cells, full-length human TSPAN4 was tagged with enhanced green fluorescent protein (EGFP) fluorescent protein at the amino terminus and cloned into the lentiviral expression vector. Following verification by Sanger sequencing and restriction digest, a stable KYSE-150-TSPAN4-EGFP cell line was generated by lentiviral transduction and followed by selection with 5 μg/ml puromycin for 48–72 h. Subsequently, the stable line was expanded and seeded into six-well plates during the logarithmic growth phase. Live cell imaging was performed using a fluorescence microscope (Nikon). KYSE-150-TSPAN4-EGFP cells were kept at 5% CO_2_ and 37°C during the experiments, and images were taken with a ×20 objective.

### Migrasome isolation

Migrasomes were isolated using the ultracentrifugation technique according to Yu’s lab protocol (https://liyu-lab-tsinghua.github.io/protocols/). The medium of the cells was replaced without serum or PS when the cells reached 80% confluence. About 48 h later, culture supernatant of hypoxic and normoxic ESCC cells was collected and subjected to sequential centrifugation at 1000 *g* for 10 min, 4000 *g* for 20 min, and 20 000 *g* for 30 min to obtain crude migrasome pellets. These pellets were washed with phosphate-buffered saline (PBS) and recentrifuged to purify them. For further purification, a stepwise Optiprep density gradient was prepared, ranging from 30% to 2% (each step 500 μl). The crude pellets were resuspended in 19% Optiprep within the gradient. Ultracentrifugation was performed at 150 000 *g* for 4 h at 4°C. Gradient fractions (480 μl each) were collected from top to bottom, diluted with PBS, and centrifuged at 20 000 g for 30 min to pellet the migrasomes.

### Transmission electron microscopy

Morphology of the migrasomes was observed under a transmission electron microscope JEM-1200EX. First, the resuspended migrasome in phosphate buffer solution was applied onto a copper electron grid. Allow the sample to settle for 1 min. Next, use filter paper to gently absorb any excess liquid from the edge of the grid. Following the above step, apply 10 μl of phosphotungstic acid to the copper grid, settle samples for 1 min, and again use filter paper to remove the excess liquid. After this step, the samples were allowed to air-dry at ambient temperature for 2 min. Once the samples are dry, they are ready for microscopic examination, operated at an acceleration voltage of 100 kV.

### Western blot

Protein from whole cell extracts was extracted by using RIPA buffer (Thermo, USA) supplemented with protease inhibitor (Roche) and boiled for 5 min in 5 × sodium dodecyl sulfate sample buffer. Protein samples were subjected to sodium dodecyl sulfate–polyacrylamide gel electrophoresis and transferred to a polyvinylidene difluoride membrane for immunoblotting analysis. The membranes were then blocked with 5% skim milk for 1 h and then incubated overnight at 4°C using the following antibodies: PIGK (Proteintech, USA), CPQ (Proteintech, USA), TSPAN4 (Invitrogen, USA), CD9 (Proteintech, USA), CD81 (Proteintech, USA), NDST1 (Proteintech, USA), CD63 (Abcam, USA), TSG101 (Abcam, USA), COL1A1 (Proteintech, USA), COL3A1 (Proteintech, USA), and glyceraldehyde-3-phosphate dehydrogenase (GAPDH) (Proteintech, USA). Secondary horseradish peroxidase-linked antibodies (Beyotime, China) were subsequently probed blots for 1 h at room temperature. Protein expression in each sample was detected using chemiluminescence imaging analysis and quantified by normalizing GAPDH levels with ImageJ software.

### RNase-protection assay

To validate that RNA is enclosed within the intact membrane of migrasomes rather than being surface-adhered, an RNase-protection assay was performed. Purified migrasomes were divided into three treatment groups: (i) negative control, (ii) digestion with RNase A (100 μg/ml, 37°C, 30 min), and (iii) digestion with RNase A (100 μg/ml) plus 0.1% Triton X-100 (37°C, 30 min). After treatment, total RNA was immediately extracted using TRIzol reagent. The levels of selected migrasomal RNAs were then quantified by RT-qPCR.

### Reverse transcription‑quantitative real-time polymerase chain reaction (RT‑qPCR)

RNA was extracted using TRIzol reagent (T9424, Sigma, USA). The aqueous phase obtained after chloroform separation was precipitated with isopropanol. The RNA pellet was washed with 75% ethanol and dissolved in RNase-free water. Concentration and purity were determined spectrophotometrically, and integrity was confirmed by agarose gel electrophoresis. cDNA was synthesized from 1 μg total RNA using random primers and reverse transcriptase (Roche, Switzerland) with deoxy-ribonucleoside triphosphate and an RNase inhibitor. The reaction proceeded at 25°C for 5 min, 42°C for 60 min, and 70°C for 5 min. RT-qPCR was performed on a LightCycler® 480 system in 10 μl reactions containing SYBR Green Master Mix, 0.3 μM primers, and cDNA. Cycling conditions were: 95°C for 10 min; 45 cycles of 95°C for 15 s, and 60°C for 60 s. Melt-curve analysis confirmed specificity. Relative expression was calculated by the 2 − ΔΔCt method using an endogenous reference gene. Primers are listed in Table S1.

### Migrasome tracing assay

The EGFP-positive migrasomes derived from KYSE-150-TSPAN4-GFP cells cultured under normoxic or hypoxic (1% O₂) conditions for 48 h were collected by ultracentrifugation. The migrasomes were labeled with wheat germ agglutinin (WGA, WGA-AF594, W11262, Thermo Fisher Scientific, USA) and then incubated with MRC-5. After incubation, cells on coverslips were washed three times with PBS (5 min each) and stained with Hoechst 33342 (C1029, Beyotime, China) for 10 min at room temperature in the dark. Following three additional PBS washes (5 min each), coverslips were mounted and observed under a fluorescence microscope to visualize WGA-labeled migrasomes and Hoechst-stained nuclei.

### Transwell assay

Cell migration and invasion capabilities were assessed using the Transwell assay. Cells were divided into three groups: PBS, Nor-mig, and Hypo-mig. Migrasomes were collected from donor cells cultured under hypoxic conditions (1% O₂) for 48 h in a tri-gas incubator and isolated by ultracentrifugation. Before the assay, MRC-5 cells were pretreated with 50 μg/ml mitomycin C (HY-13316, MCE, USA) for 2 h to inhibit proliferation, then detached, counted, and adjusted to 2 × 10^4^ cells/ml in serum-free MEM. For the migration test, MRC-5 cells in 500 μl serum-free minimum essential medium (MEM) containing 100 μg/ml of the corresponding migrasome preparation were placed in the upper chamber of an 8.0 μm Transwell insert (353097, FALCON, USA), while 700 μl MEM with 10% FBS was added to the lower chamber. After a 48-h incubation at 37°C, the upper chamber was fixed with 4% paraformaldehyde for 30 min and stained with crystal violet for the same duration. The stained cells were then mounted on slides and sealed with neutral balsam, and three random fields from both central and peripheral areas of the membrane were analyzed using ImageJ software for cell counting. For the invasion test, Matrigel-coated  8.0μm Transwell inserts (354480, BioCoat, USA) were utilized, with all other procedures following the same steps as the migration assay.

### Enzyme‑linked immunosorbent assay (ELISA)

ELISA analysis of IL-1β and TGF-β levels in the fibroblast conditional medium (CM) samples was performed using IL-1β ELISA Kit (ml058059, Mlbio, China) and active TGF-β ELISA Kit (ml064258, Mlbio, China), following the manufacturer’s instructions. The CM was kept undiluted. After the experiment was terminated, the optical density (OD) values of each well were measured at a wavelength of 450 nm.

### Immunofluorescence staining

For immunofluorescence staining, cells were seeded and treated on coverslips as described. After treatment, cells were washed once with PBS and fixed with 4% paraformaldehyde for 20 min at room temperature. Following three PBS washes (5 min each), cells were permeabilized with 0.1% Triton X-100 for 5 min and washed again. Nonspecific binding was blocked with 3% BSA for 30 min. Cells were then incubated with anti-α-SMA antibodies (14395-1-AP, Proteintech, USA) diluted 1:1000 in PBS at 4°C overnight. After washing, cells were incubated with corresponding fluorescence-conjugated secondary antibodies (1:500 dilution in PBS; ab150077, Abcam, USA) for 1 h at room temperature in the dark. Nuclei were stained with DAPI (C1006, Beyotime, China) for 10 min. Coverslips were mounted and visualized under a fluorescence microscope.

### Whole-transcriptome sequencing

Trizol reagent was used to extract the total RNA from normoxic and hypoxic migrasomes. The RNA purity and concentration were confirmed using a NanoDrop ND-1000 spectrophotometer (Thermo Scientific, USA), and the integrity was determined by agarose gel electrophoresis. Each condition (normoxia and hypoxia) included three biological replicates (*n* = 3 per group). The RNA-seq libraries were prepared from isolated total RNA from normoxic and hypoxic migrasomes by TruSeq Stranded Total RNA Sample Preparation kit (Illumina, USA). Ribosomal RNA (rRNA) was depleted from extracted total RNA by using target-specific oligos and RNase H reagents. The cleaved RNA fragments were then reverse-transcribed into cDNA using reverse transcriptase (Invitrogen, USA) and random primers. Final cDNA libraries were subjected to paired-end sequencing on the Illumina HiSeq2000 platform (Illumina, USA) following the manufacturer’s protocol.

Clean reads were aligned to the reference genome. mRNAs and lncRNAs were annotated based on existing genomic annotations, with no additional filtering criteria applied. A gene was considered expressed and retained for subsequent analysis if it had a raw count >5 in at least one sample. circRNAs were identified from the aligned data using the ACFS2 pipeline based on reliable back-splicing junction evidence. Differential expression analysis for each RNA category was performed using raw read counts as input to DESeq2. Given the limited RNA input and exploratory nature, differential expression was defined as |log₂FC| > 1 and nominal *P* <0 .05 (exploratory dataset). No genes passed false discovery rate (FDR) < 0.05 due to low statistical power, so a high-confidence dataset could not be defined. The host genes of differentially expressed (DE) RNAs were mapped to terms in the Gene Ontology (GO) database through GO enrichment analysis, employing the R package “clusterProfiler” (*P* < .05). GO enrichment analysis was performed based on this gene set and interpreted as exploratory. Additionally, the Kyoto Encyclopedia of Genes and Genomes (KEGG) pathway enrichment analysis was utilized to ascertain their biological functions, also facilitated by the R package “clusterProfiler” (|log₂FC| > 1, *P*-value < .05). KEGG pathway analysis was conducted in an exploratory manner, with results interpreted as indicative of potential biological trends.

### Prediction of ceRNA regulatory network

Potential interactions between miRNAs and mRNAs, lncRNAs, or circRNAs were predicted using the miRanda and RNAhybrid algorithms. For miRanda, strict seed matching was enforced (Seed_Strict = TRUE) with thresholds of score ≥ 150 and free energy ΔG ≤ −20 kcal/mol. For RNAhybrid, the human model was applied with a maximum of 200 000 targets per miRNA, up to 50 binding sites per target, and a free energy threshold of ΔG ≤ −25 kcal/mol. Only interactions identified by both tools were retained as high-confidence miRNA–RNA pairs.

To infer ceRNA relationships, miRNAs that were predicted to target both a differentially expressed lncRNA (or circRNA) and a differentially expressed mRNA were selected. Furthermore, only lncRNA–mRNA (or circRNA–mRNA) pairs that showed concordant expression changes were included in the final ceRNA network.

### Statistical analysis

Quantitative data are presented as the means ± standard deviations (SDs) from at least three independent experiments and analyzed by GraphPad Prism 9.0. The differences, including more than two groups, were presented by one-way analysis of variance (ANOVA). Comparisons for two groups were performed by a two-tailed *t-*test. A significant difference was represented when *P* < .05.

## Results

### Characterization of hypoxic migrasomes from esophageal squamous cell carcinoma cells

**Figure 1:**
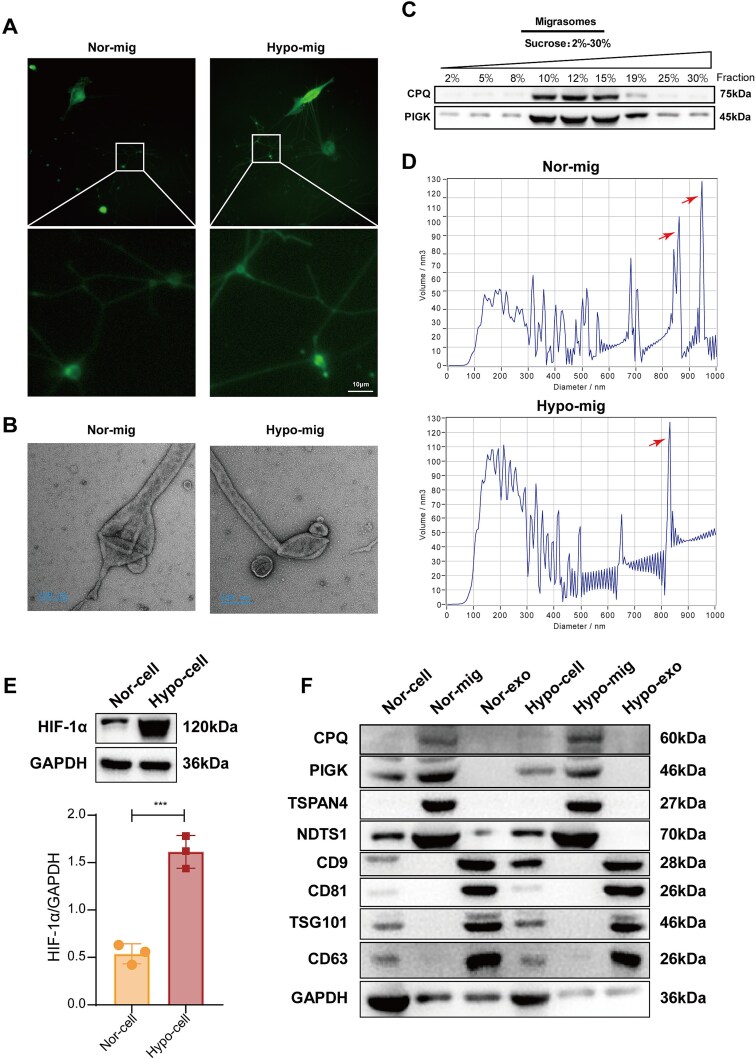
Characterization of hypoxic migrasomes from ESCC cells. (A) Representative fluorescence images of the KYSE-150 cell–derived migrasomes revealed by a fluorescence microscope using TSPAN4-EGFP fusion plasmid. Images were acquired at 40× magnification. Scale bars: 10 μm. (B) TEM was used to observe normoxic and hypoxic migrasomes. Scale bars: 200 nm. (C) Western blot analysis of migrasome markers (CPQ, PIGK) across OptiPrep density-gradient fractions (2%–30%) from extracellular vesicles. (D) Nanoparticle tracking analysis (NTA) showing the size distribution of migrasomes isolated under normoxia and hypoxia. (E) Western blot confirming HIF-1α induction in KYSE-150 cells cultured under hypoxia before migrasome isolation. The corresponding densitometric quantification (HIF-1α/β-actin) is presented as mean ± SD (*n* = 3). (F) Extended marker panel analysis by western blot demonstrating enriched expression of migrasome-associated proteins (CPQ, PIGK, TSPAN4, NDST1) and absence of detectable exosome markers (CD9, CD81, TSG101, CD63) in isolated migrasomes. ^***^P < .001 by unpaired two-tailed *t-*test.

Migrasomes have been identified in various tumor cells, but the effect of tumor hypoxic microenvironment on them has not been clear. To explore whether ESCC cells with intense aggressiveness also produce migrasomes at the cell posterior margin and whether hypoxia affects them, we utilized TSPAN4-EGFP fusion plasmid to construct the stable KYSE-150-TSPAN4-EGFP cell line, followed by culturing under normoxic and hypoxic conditions. A fluorescence microscope revealed that a typical migrasomal structure, characterized by large vesicles on one side of the ESCC cells, formed on the tips or branch points of the RFs by TSPAN4 expression. However, hypoxia had no significant effect on the distribution and number of vesicles (Fig. 1A). The ultrastructure of vesicles was further observed by TEM, as indicated by round bodies binding to RFs with diameters of ~2 μm (Fig. 1B). There was no obvious difference in the morphological and structural characteristics between corresponding amounts of normoxic and hypoxic migrasomes. To validate vesicle identity and purity, isolated migrasomes were subjected to OptiPrep density-gradient ultracentrifugation. Western blot analysis showed that the migrasome markers CPQ and PIGK were predominantly enriched in the 10%–15% density fractions in both groups (Fig. 1C), consistent with reported migrasome buoyant densities. Nanoparticle tracking analysis revealed comparable size distributions, with major peaks at ~800–1000 nm under both normoxic and hypoxic conditions (Fig. 1D), a range consistent with previously reported migrasome sizes [22]. To validate the effectiveness of the hypoxic model, we first examined the induction of HIF-1α in ESCC cells exposed to 1% O₂. Western blot analysis showed a marked increase in HIF-1α protein levels in hypoxia-treated cells compared with normoxic cells, confirming robust hypoxic activation prior to migrasome isolation (Fig. 1E). In addition, an expanded panel of vesicle markers demonstrated robust expression of migrasome-associated proteins (CPQ, PIGK, TSPAN4, NDST1), whereas classical exosome markers (CD9, CD81, TSG101, CD63) were undetectable (Fig. 1F). Collectively, ESCC cells can produce RFs and migrasomes, and hypoxia has no significant effect on the amount and morphology of migrasomes.

### Global transcriptomic profiling of migrasome RNA cargo

Sequencing of the six migrasome-derived RNA libraries generated a total of 524.4 million clean paired-end reads, with individual samples yielding 63.6–112.2 million reads, providing sufficient coverage for whole-transcriptome analysis (Table S2). The overall mapping rates to the reference genome were relatively low (1.1%–2.6%), which may be attributed to the intrinsic biological and technical characteristics of migrasome-derived RNA, including a high proportion of fragmented RNA species, noncoding RNAs, and potentially unannotated transcripts, as well as limited representation of these RNA species in current reference genome annotations.

To assess data reproducibility and transcriptomic changes under hypoxia, we performed principal component analysis (PCA) and MA plots for all detected mRNAs, lncRNAs, and circRNAs. PCA plots revealed subtle but detectable separation between normoxic and hypoxic conditions, with biological replicates clustering tightly within each group, confirming experimental consistency despite the typically low RNA content of migrasomes (Fig. S1A, C, and  E).

### DEmRNA profile between normoxic and hypoxic migrasomes

**Figure 2:**
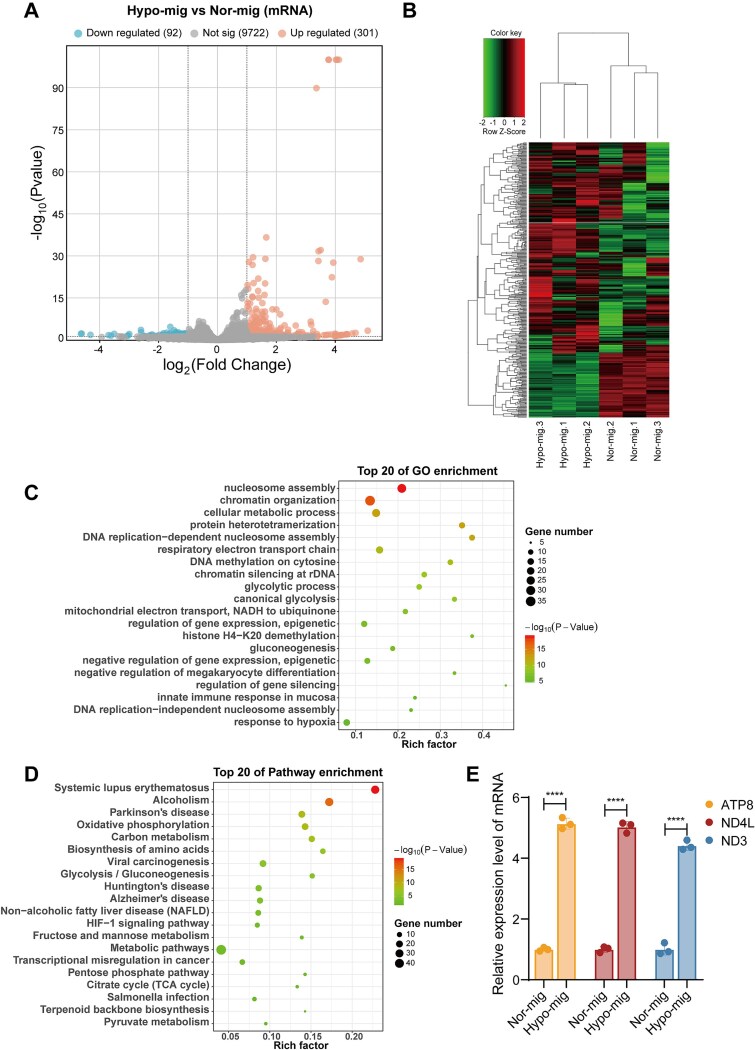
Differentially expressed (DE) mRNA profile between normoxic and hypoxic migrasomes. (A) The volcano plot displayed the DEmRNAs between normoxic migrasomes and hypoxic migrasomes. (B) The heatmap of DEmRNAs between normoxic migrasomes and hypoxic migrasomes. (C) GO enrichment analysis of host genes for DEmRNAs. (D) KEGG enrichment analysis of host genes for DEmRNAs. (E) RT-qPCR validation of representative DE mRNAs (ATP8, ND4L, ND3). Data are presented as mean ± SD (*n* = 3). ^****^P < .0001 by unpaired two-tailed *t-*test.

To explore whether hypoxia plays a role in affecting the composition of the migrasomes, we investigated the total RNA profile of migrasomes *via* whole-transcriptome sequencing. Volcano plot and MA plot analyses revealed 393 DEmRNAs (|log₂FC| > 1, *P*-value < .05), of which 301 were up-regulated and 92 were down-regulated in the hypoxic migrasomes compared with the normoxic group (Fig. 2A; Fig. S1B). The top 10 DEmRNAs are listed in Table S3, which includes upregulated mitochondrial genes such as ATP8, ND4L, and ND3. Distinguishable DEmRNAs expression patterns were identified among the migrasome samples by heatmap (Fig. 2B). Subsequently, we performed enrichment analysis to explore the potential functions and signal pathways of DEmRNAs. GO enrichment analysis revealed that DEmRNAs were significantly enriched in “response to hypoxia,” “glycolytic process,” and “regulation of gene expression, epigenetic” (Fig. 2C). KEGG pathway analysis showed that DEmRNAs were related to “transcriptional misregulation in cancer,” “glycolysis/gluconeogenesis,” and “HIF−1 signaling pathway” (Fig. 2D), suggesting that the DEmRNAs may play a key role in cancer cell adaptation to a hypoxic microenvironment. In addition, the DEmRNAs in hypoxic migrasomes were involved in functions related to “DNA methylation,” “gene silencing,” “glycolytic process,” and “cellular metabolic process” biological processes (Fig. 2C), and the pathways related to energy metabolism such as “biosynthesis of amino acids”, “glycolysis/gluconeogenesis”, “citrate cycle (TCA cycle),” and “pyruvate metabolism” pathways were also enriched (Fig. 2D). To validate the sequencing data, representative DE mRNAs were selected based on FDR < 0.05 and top three upregulated fold changes, including ATP8, ND4L, and ND3, and their expression levels were confirmed by RT-qPCR, showing low expression in normoxic migrasomes and high expression in hypoxic migrasomes (Fig. 2E). These findings suggested that hypoxia influences the RNA cargo of migrasomes and mediates intricate gene regulatory networks in ESCC.

### Transcriptional landscape and predictive potential of lncRNAs

**Figure 3:**
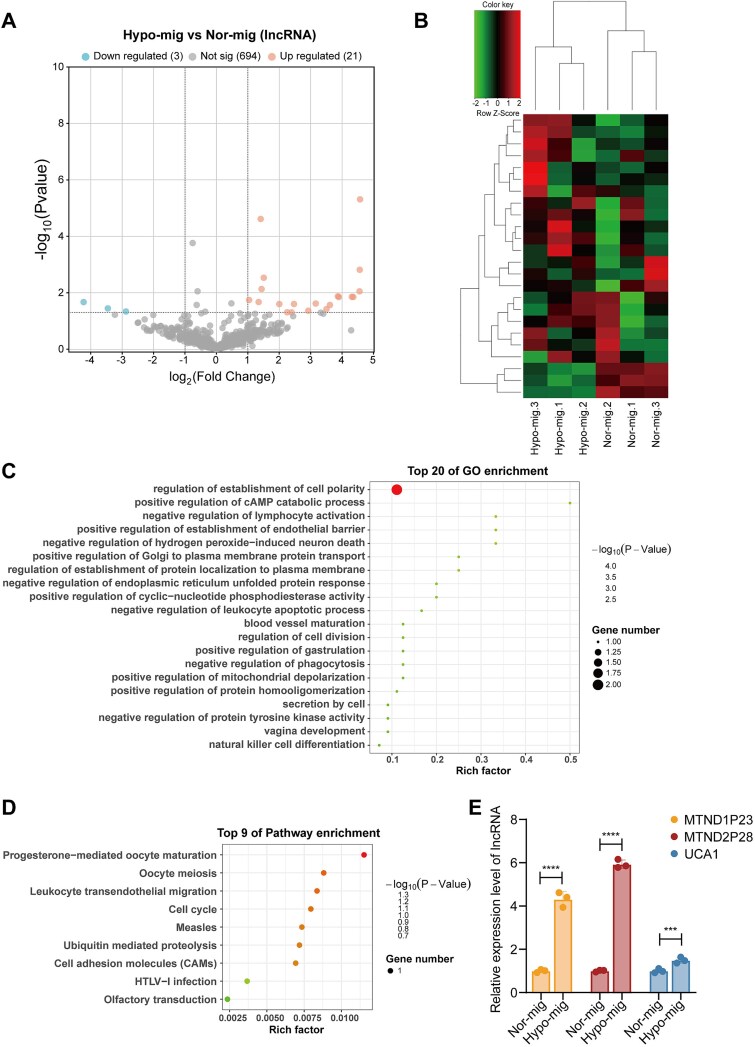
Function and signaling pathway analysis of DElncRNAs. (A) The volcano plot displayed the DElncRNAs between normoxic migrasomes and hypoxic migrasomes. (B) The heatmap of DElncRNA between normoxic migrasomes and hypoxic migrasomes. (C) GO enrichment analysis of host genes for DElncRNA. (D) KEGG enrichment analysis of host genes for DElncRNA. (E) RT-qPCR validation of representative DE lncRNAs (MTND1P23, MTND2P28, UCA1). Data are shown as mean ± SD (*n* = 3). ^***^P < .001, ^****^P < .0001 by unpaired two-tailed *t-*test.

LncRNA is a noncoding RNA with a length between 200 and 100 000 nucleotide units [23]. Growing evidence has elaborated that lncRNAs gain particular relevance in tumor cell proliferation, metastasis [24], drug resistance [25], and immune evasion [26] through the transmission of intercellular signals by extracellular vesicles. However, it is not clear whether the migrasomes are able to carry lncRNAs. Here, we profiled the DElncRNAs between normoxic and hypoxic migrasomes by whole-transcriptome sequencing. Compared with the normoxic migrasomes, we found 24 DElncRNAs (21 up-regulated, 3 down-regulated) in the hypoxic migrasomes *via* volcano plot and MA plot analyses (|log₂FC| > 1, *P*-value < .05) (Fig. 3A; Fig. S1D). The top 10 DElncRNAs are listed in Table S4, which includes genes such as MTND2P28, MTND1P23, and UCA1. Cluster analysis revealed distinct clusters between normoxic and hypoxic migrasomes (Fig. 3B). We predicted the target genes of DElncRNAs based on the ceRNA mechanism. GO analyses were performed to identify functional enrichment of DElncRNA target genes, which were most highly enriched for terms such as “establishment of cell polarity,” “establishment of protein localization to plasma membrane,” and “golgi to plasma membrane protein transport” (Fig. 3C). Additionally, DElncRNAs were involved in the processes including “cAMP catabolic process” and “cyclic-nucleotide phosphodiesterase activity,” “endoplasmic reticulum unfolded protein response,” and “mitochondrial depolarization.” The KEGG pathway enrichment analysis indicated that the DElncRNAs were involved in “cell adhesion molecules,” “cell cycle,” and “ubiquitin-mediated proteolysis” (Fig. 3D). Representative DE lncRNAs, including MTND1P23, MTND2P28, and UCA1, were further validated by RT-qPCR. These lncRNAs were selected based on the top three upregulated fold changes in hypoxic migrasomes, and the RT-qPCR confirmed low expression in normoxic migrasomes and high expression in hypoxic migrasomes (Fig. 3E). These data indicated that the hypoxia microenvironment altered the transcriptome of lncRNAs, which presented the likelihood of involvement of processes such as cell polarity, metabolic pathways, and ubiquitination modification, further affecting the TME.

### Global dysregulation of circRNAs

**Figure 4:**
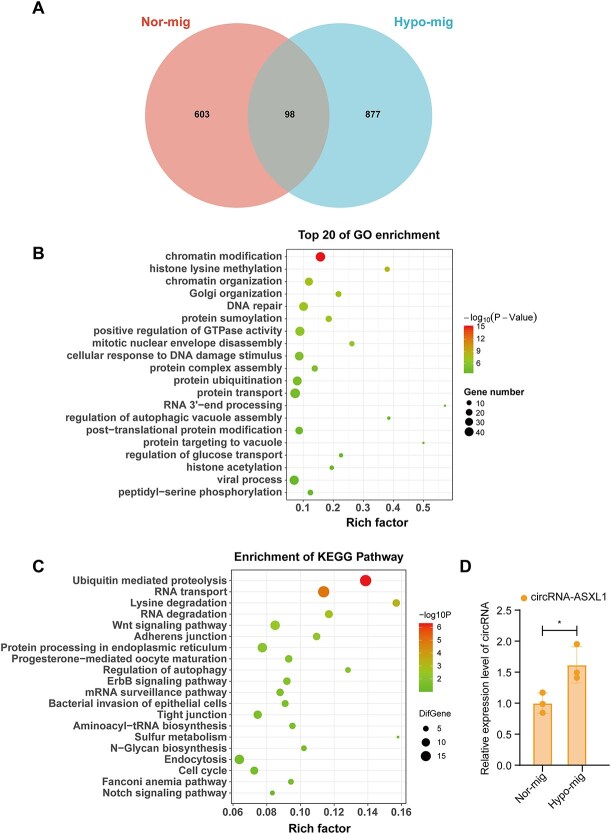
Enrichment analysis of DEcircRNAs. (A) The Venn diagram displayed the overlap of circRNAs between normoxic and hypoxic migrasomes. (B) The top 20 enriched GO pathways of the hypoxic circRNA host genes. (C) The enriched KEGG pathways of the circRNA host genes. (D) RT-qPCR validation of representative circRNAs - chr20_32369123_32366384_ + 2739-ASXL1. Data are presented as mean ± SD (*n* = 3). ^*^P < .05 by unpaired two-tailed *t-*test.

Circular RNAs (circRNAs) are a class of highly conserved regulatory ncRNAs that arise from the back-splicing of exons, introns, or intergenic regions [27]. In esophageal cancer, circRNAs extensively affect cancer cell proliferation, metastasis, apoptosis, and metabolism [28, 29]. Compared with mRNA and lncRNA in ESCC migrasomes, circRNAs were of low abundance. Specifically, expression of 98 circRNAs (counts >0 in at least one sample) was common in hypoxic and normoxic migrasomes; 603 and 876 circRNAs were expressed by normoxic and hypoxic migrasomes, respectively (Fig. 4A). To investigate the functions of circRNAs in hypoxic migrasomes, we analyzed the host genes of hypoxic migrasomal circRNAs. GO enrichment analysis revealed that the host genes of hypoxic circRNAs were mainly enriched in “ubiquitin binding” and the “ubiquitin-dependent protein catabolic process” (Fig. 4B). Ubiquitination has been reported to activate CAFs and participate in tumor progression [30]. These data suggested that circRNAs may activate CAFs by mediating protein ubiquitination. In addition, significant GO enrichment was identified for genomic instability-related pathways, such as “chromatin remodeling,” “DNA damage response,” “DNA repair,” and “positive regulation of transcription by RNA polymerase II.” circRNA can promote cancer development by regulating cell chromatin remodeling and damage repair function of DNA double-strand breaks [31]. Oxidative stress–sensitive circMdc1 regulated DNA damage and chromosome stability in cardiomyocytes by blocking the translation of the host gene Mdc1 [32]. The results of the KEGG pathway enrichment analysis illustrated that migrasomal circRNAs might influence the “Notch signaling pathway” and “Wnt signaling pathway” (Fig. 4C), which were known to be activated in CAFs [33, 34]. MA plot analysis demonstrated that only one differentially expressed circular RNA (DEcircRNA) was identified under the screening thresholds of |log₂FC| > 1 and *P*-value < .05 (Fig. S1F;  Table  S5). We further validated the DEcircRNA, chr20_32369123_32366384_ + 2739-ASXL1 by RT-qPCR, confirming low expression in normoxic migrasomes and high expression in hypoxic migrasomes (Fig. 4D). These data suggested that hypoxia-induced migrasomal circRNAs may be involved in CAF activation and the ESCC process through regulation of genomic instability and ubiquitination modification.

### CeRNA network construction

**Figure 5:**
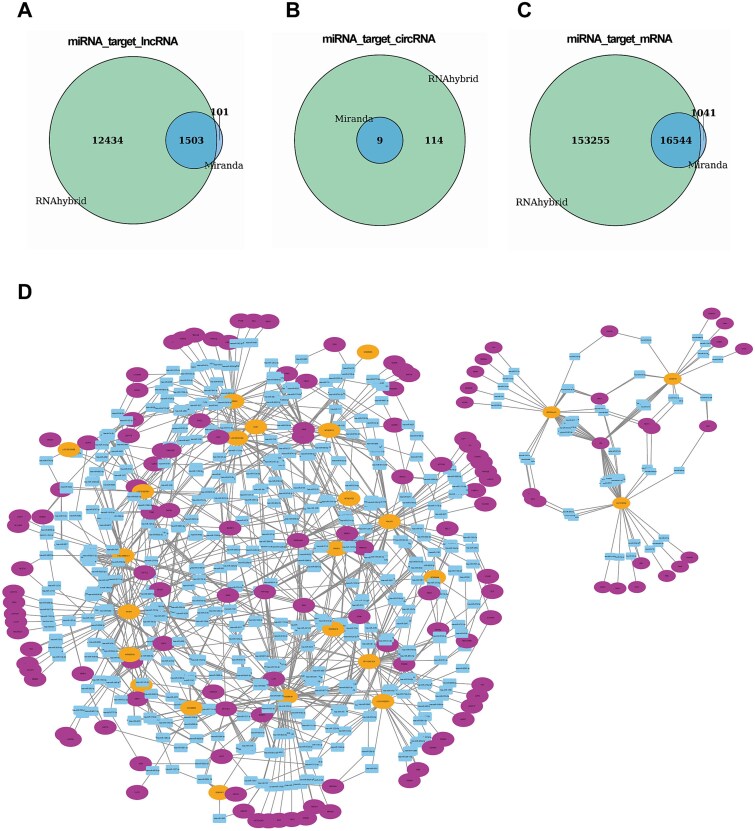
Intersection of miRNA targeting predictions for three RNA types and competing endogenous RNA (ceRNA) network integration. (A) Venn diagram of common miRNAs targeting lncRNAs predicted by both RNAhybrid and Miranda. (B) Venn diagram of common miRNAs targeting circRNAs predicted by both RNAhybrid and Miranda. (C) Venn diagram of common miRNAs targeting mRNAs predicted by both RNAhybrid and Miranda. (D) The ceRNA Network. Orange nodes represent DElncRNAs. Purple nodes represent mRNAs. Blue nodes represent DEmiRNAs.

A well-established mechanism by which miRNAs modulate target gene expression involves the ceRNA network. To explore the molecular pathways through which ESCC-derived migrasomes regulate CAFs, we systematically constructed a ceRNA regulatory network. In this study, we employed the RNAhybrid and Miranda algorithms to predict miRNA interactions with DElncRNAs, circRNAs, and mRNAs. Requiring consensus predictions from both tools, we identified 1503 miRNA-targeted lncRNAs, 9 miRNA-targeted circRNAs, and 16 544 miRNA-targeted mRNAs (Fig. 5A–c). Subsequently, by screening for circRNAs, lncRNAs, and mRNAs co-regulated by shared miRNAs, we constructed a competing endogenous RNA (ceRNA) network (Fig. 5D). ceRNA networks were established by identifying circRNAs, lncRNAs, and mRNAs sharing common miRNA response elements (Fig. 5D). The resulting exploratory network comprised 659 miRNAs (e.g*.* hsa-miR-145-5p, hsa-miR-424-5p), 24 lncRNAs (e.g. SERTAD4-AS1, LINC00920), and 132 mRNAs (e.g. ICAM1, KLF6, TGM2). Notably, ICAM1—identified within this predicted network—has been demonstrated to promote inflammation-dependent extracellular matrix contraction, facilitating cancer cell invasion [35], providing a plausible biological context for the network predictions. Importantly, this ceRNA network is prediction-based and lacks direct functional validation; therefore, the inferred regulatory relationships should be interpreted with caution and considered exploratory.

### Hypoxic migrasomes from esophageal squamous cell carcinoma cells activate the cancer-associated fibroblasts

**Figure 6:**
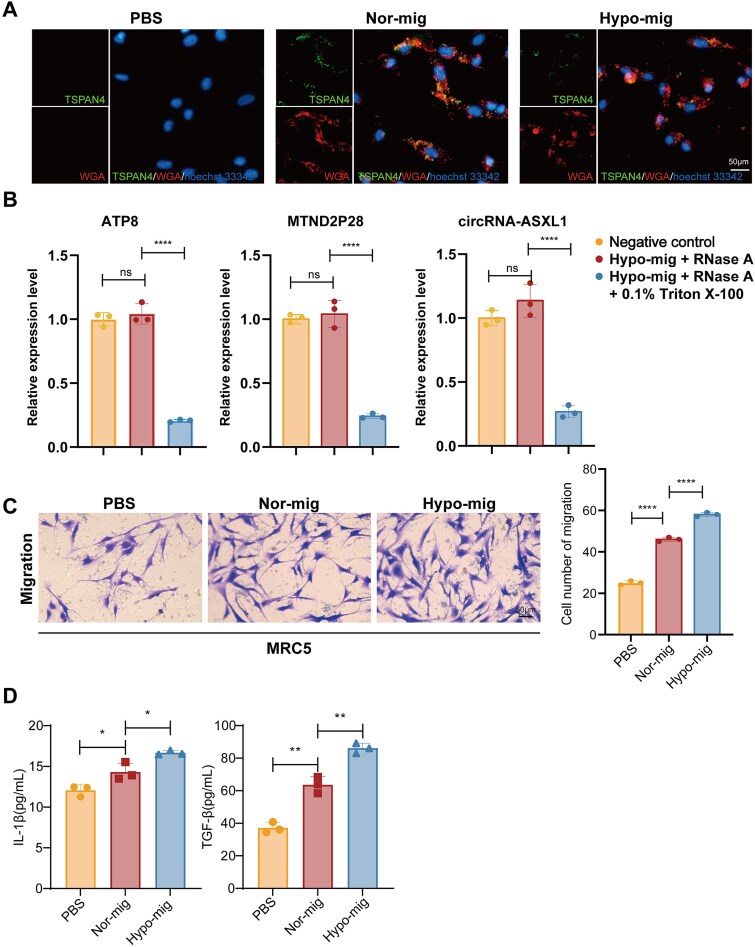
Hypoxic migrasomes from ESCC cells are internalized by fibroblasts and activate the CAFs. (A) Internalization of ESCC cell-derived migrasomes by fibroblasts, demonstrated by co-localization (yellow) of TSPAN4-EGFP-labeled migrasomes (green) with WGA staining (red) of fibroblast membranes. Nuclei were counterstained with Hoechst 33342 (blue). Images were acquired at 40× magnification. Scale bar: 50 μm. (B) RNase-protection assay confirms RNA cargo is enclosed within intact migrasomes. Purified migrasomes were treated under three conditions: (1) negative control, (2) RNase A, or (3) RNase A + 0.1% Triton X-100. Total RNA was then extracted, and the levels of representative migrasomal RNAs (ATP8, MTND2P28, and circRNAs - chr20_32369123_32366384_ + 2739-ASXL1) were quantified by RT-qPCR. Data are presented as mean ± SD (*n* = 3). (C) Representative images and quantification of Transwell migration assays. MRC-5 fibroblasts, pretreated with mitomycin C, were incubated with PBS, Nor-mig, or Hypo-mig. Images were acquired at 20× magnification. Scale bar: 50 μm. Data are shown as mean ± SD (*n* = 3). (D) Hypoxic migrasomes enhance IL-1β and active TGF-β secretion by fibroblasts. ELISA quantification of (left) IL-1β and (right) TGF-β in fibroblast conditioned medium after treatment with PBS, Nor-mig, or Hypo-mig. Data are mean ± SD (*n* = 3). Exact concentrations (pg/ml): IL-1β – PBS: 12.05 ± 0.68, Nor-mig: 14.31 ± 1.09, Hypo-mig: 16.67 ± 0.26. TGF-β – PBS: 37.18 ± 3.20, Nor-mig: 63.59 ± 5.00, Hypo-mig: 86.15 ± 3.08. ns, not significant; ^*^P < .05, ^**^P < .01, ^****^P < .0001 (analyzed by unpaired two-tailed *t-*test and one-way ANOVA).

**Figure 7:**
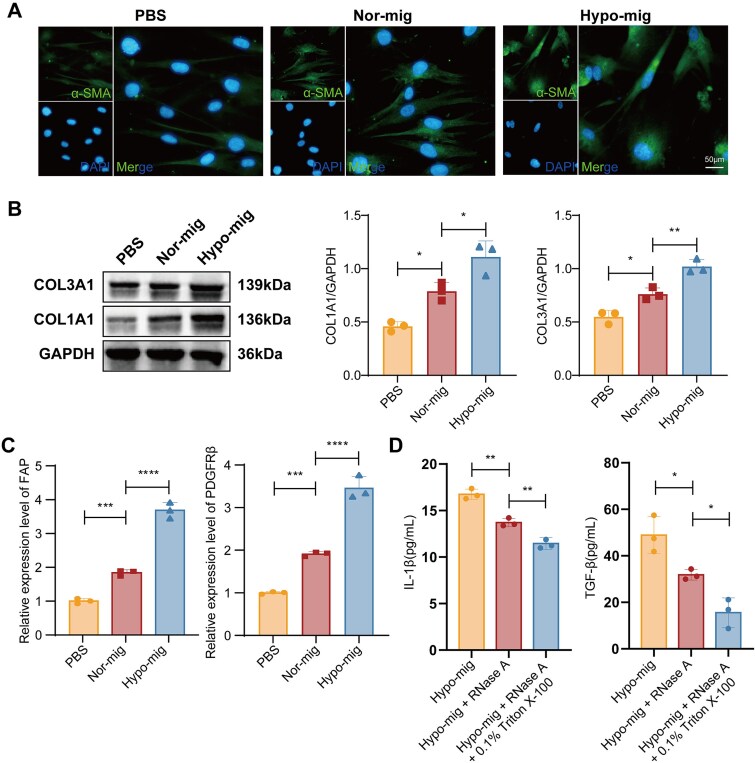
Hypoxic migrasomes induce a CAF-like phenotype in fibroblasts. (A) Immunofluorescence detection of α-SMA in fibroblasts. Images were acquired at 40× magnification. Scale bar: 50 μm. (B) Western blot analysis of COL1A1 and COL3A1 in MRC-5 cells. Data are shown as mean ± SD (*n* = 3). (C) RT-qPCR detection of CAF markers FAP and PDGFRβ in fibroblasts. Data are shown as mean ± SD (*n* = 3). (D) ELISA quantification of IL-1β and TGF-β secretion by fibroblasts after co-culture with hypoxic migrasomes pretreated as follows: (1) intact Hypo-Mig, (2) Hypo-Mig + RNase A, and (3) Hypo-Mig + RNase A + 0.1% Triton X-100. Data are mean ± SD (*n* = 3). Exact concentrations (pg/ml): IL-1β – intact Hypo-Mig: 16.41 ± 0.55, Hypo-Mig + RNase A: 13.70 ± 0.44, Hypo-Mig + RNase A + Triton X-100: 11.45 ± 0.62; TGF-β – intact Hypo-Mig: 48.90 ± 7.9, Hypo-Mig + RNase A: 31.81 ± 2.3, Hypo-Mig + RNase A + Triton X-100: 15.56 ± 6.3. ^*^P < .05, ^**^P < .01, ^***^P < .001, ^****^P < .0001 by one-way ANOVA.

Given that migrasome production is a critical mechanism for the communication between cancer cells and the TME, we further investigated whether hypoxic migrasomes participate in the activation of fibroblasts. First, we evaluated whether ESCC cell–derived migrasomes could be ingested by fibroblasts. We confirmed internalization by co-localization of TSPAN4-EGFP-labeled migrasomes with WGA staining in fibroblasts (Fig. 6A), indicating that ESCC cell–derived migrasomes were effectively taken up by fibroblasts. To determine whether the identified RNAs are enclosed within the migrasomes rather than being surface‑adhered, we performed an RNase protection assay. Isolated hypoxic migrasomes were divided into three treatment groups: (i) negative control, (ii) RNase A, and (iii) RNase A + 0.1% Triton X-100. The levels of three key hypoxia-upregulated RNAs—ATP8, MTND2P28, and the circRNA chr20_32369123_32366384_ + 2739-ASXL1—were then quantified by RT-qPCR. The results showed that RNase A treatment alone did not significantly reduce RNA levels, whereas co-treatment with Triton X-100 led to a sharp decrease (Fig. 6B). In the CAF-activated phenotype, the upregulation of pro-inflammatory cytokines in fibroblasts is accompanied by the acquisition of enhanced migratory ability. To assess this, MRC-5 fibroblasts were incubated with PBS, normoxic migrasomes (Nor-mig), or hypoxic migrasomes (Hypo-mig). Our results demonstrated that the addition of hypoxic migrasomes significantly enhanced fibroblast migration compared to the Nor-mig group (Fig. 6C). We detected the concentrations of pro-inflammatory cytokines in the supernatant using an ELISA, revealing hypoxic migrasomes induced a concurrent increase in IL-1β and TGF-β secretion compared with normoxic migrasome and PBS groups (Fig. 6D). To further confirm fibroblast activation into CAFs, we assessed classical CAF markers. Immunofluorescence staining for α-SMA showed a marked increase in α-SMA-positive fibroblasts upon hypoxic migrasome treatment compared with normoxic migrasome and PBS groups (Fig. 7A). Consistent with these findings, western blot analysis showed that hypoxic migrasomes also augmented the expression of collagens COL1A1 and COL3A1 (Fig. 7B). Additionally, RT-qPCR analysis revealed that FAP and PDGFRβ expression levels were low in PBS-treated fibroblasts, moderately increased in normoxic migrasome-treated fibroblasts, and highest in hypoxic migrasome-treated fibroblasts (Fig. 7C). To determine whether the functional RNA cargo within migrasomes was required for CAF activation, hypoxic migrasomes were pretreated with RNase A alone or in combination with the membrane-disrupting agent Triton X-100 prior to co-culture with fibroblasts. ELISA analysis revealed that the robust induction of IL-1β and TGF-β by intact hypoxic migrasomes was substantially attenuated upon RNase treatment following membrane permeabilization (Fig. 7D), indicating that the internal RNA cargo is essential for the cytokine-inducing capacity of hypoxic migrasomes. Collectively, these data suggest that hypoxic migrasomes induced a CAF phenotype in normal fibroblasts within the TME.

## Discussion

ESCC is a malignant tumor with high morbidity, recurrence, and mortality rates worldwide [1]. Owing to the complex and continuously evolving TME, it is difficult to achieve a cure for ESCC tumors [36]. Gaining insights into the biology of TME may uncover new therapeutic targets for ESCC. In this study, we first showed that ESCC cells could generate migrasomes, and hypoxia had no effect on the morphology and size of migrasomes but changed the mRNA, lncRNA, and circRNA components of the migrasomes. Hypoxia-induced migrasomal DEmRNAs were mainly associated with gene regulation and energy metabolism; DElncRNAs were implicated in cyclic adenosine monophosphate (cAMP) pathways and mitochondrial function; and DEcircRNAs were linked to genomic instability and ubiquitination modification. Furthermore, hypoxic migrasomes were found to induce the activation of normal fibroblasts into CAFs. This study reveals a novel interaction mechanism between ESCC cells and the TME, highlighting the role of hypoxic migrasomes in CAF activation and offering new strategies for targeted therapies.

Migrasomes are newly discovered single-membrane vesicular structures observed in migrating cells, including tumor cells and neutrophils. Recent studies have revealed that migrasomes serve as an intercellular communication mechanism, delivering chemokines, cytokines, and angiogenic factors to promote angiogenesis, invasion, metastasis, and proliferation in various cancers [16]. A pan-cancer analysis at the single-cell level determined that migrasome was positively correlated with hypoxia in various cancers [37]. A solid TME is characterized by oxygen depletion due to the hyperactive metabolism of tumor cells [38]. Hypoxia seems to be an influential factor in cellular communication between malignant cells and the TME. Hypoxic exosomes mediated crosstalk between tumor cells and macrophages by delivery of circRNA PLEKHM1 to promote lung cancer metastasis [39]. However, it remains unclear whether hypoxia affects the morphology, size, and contents of migrasomes, as well as their functions. In this study, we found that hypoxia had no significant effect on the amount, size, and morphology of migrasomes, but it did alter their RNA composition.

Yu *et al.* found that RNA was enriched in the detached migrasomes from mice [40]. To explore the effect of hypoxic migrasomes on the ESCC TME, we characterized the mRNA, lncRNA, and circRNA in normoxic and hypoxic migrasomes by RNA-seq. Enrichment analysis showed that the DEmRNAs between normoxic and hypoxic migrasomes were related to the pathway response to hypoxia, gene regulation including “DNA methylation” and “gene silencing,” and energy metabolism such as “biosynthesis of amino acids” and “glycolysis/Gluconeogenesis.” Recent research has revealed that hypoxic microenvironment can determine the phenotypic plasticity and spatial distribution of CAF [41]. RNA-seq found that CAFs undergo distinctive transcriptome transition in lung cancer brain metastasis under a hypoxic situation, which is directly driven by hypoxia-induced HIF signaling [42]. Hypoxia induces epigenetic reprogramming of normal fibroblasts, resulting in an abnormal glucose metabolism during the progression of breast cancer [10]. In addition, CAFs in co-culture with colon cancer organoids were characterized by glycolysis, hypoxia, and genes involved in immunosuppression [43]. Migrasomal mRNA can be expressed in recipient cells [44], suggesting that the hypoxia migrasomes from ESCC cells may regulate the metabolic process of CAF by delivering mRNA. DElncRNA between normoxia and hypoxia migrasomes are enriched in “cAMP catabolic process,” “mitochondrial depolarization,” and “ubiquitination modification.” Breast cancer cells triggered aerobic glycolysis by activating the cAMP/PKA pathway in CAFs [45], suggesting that hypoxic migrasomes enriched DElncRNAs may promote CAFs by mediating cAMP catabolic. The mitochondrial stress and endoplasmic reticulum stress are more pronounced in CAFs and seem to be associated with increased CAF activity and cellular autophagy [46]. In the CAF of skin basal cell carcinoma and squamous cell carcinoma, the heterogeneity level of mtDNA CD is reduced compared to NFs, reflecting that cancer activation may reprogram coding mtDNA to maintain mitochondrial homeostasis [47]. A stromal lncRNA-mediated ubiquitination could reprogram NFs to CAFs, promoting oral cancer development [48]. These data implied that a hypoxic environment might have affected cAMP pathways and mitochondrial function within the activated CAFs. Migrasomal circRNAs presented low abundance; we investigated hypoxia-specific circRNAs related to “DNA damage,” “chromatin remodeling,” and “ubiquitination modification.” circFARP1 blocked the interaction of CAV1 and the E3 ubiquitin-protein ligase to inhibit CAV1 ubiquitination degradation, leading to pancreatic cancer progression and poor patient survival [30]. The above results indicate that hypoxia may affect the genomic stability of cells in the ESCC TME and potentially influence CAF phenotype through protein ubiquitination modification. Our study revealed that hypoxia may mainly mediate the change of migrasomes components and participate in the regulation of TME.

Hypoxia shapes the landscape of the TME into a protumorigenic and prometastatic niche, which could be direct or indirect, either by directly inducing changes in cellular metabolism or creating a crosstalk between tumor and stromal cells [49]. Among all the stromal cells, CAFs share the most intricate relationship with cancer cells within the TME and participate in tumor genesis, development, and drug resistance by secreting various factors and exosomes [50]. A series of large-scale scRNA-seq datasets displayed that HIF1A, a master transcriptional regulator of the cellular response to hypoxia, was commonly activated in CAFs compared to normal fibroblasts [41]. However, the mechanism by which hypoxia induces CAF activation is still unknown. Pancreatic cancer–derived migrasomes could induce the cancer-promoting phenotype of stromal cells, which promotes tumor progression by contributing to an immunosuppressive TME [51]. We found that hypoxic migrasomes were uptaken by fibroblasts, which further promoted migration ability and increased the pro-inflammatory cytokines of fibroblasts. These data demonstrated that hypoxic migrasomes from ESCC cells induced the transition of normal fibroblasts into activated CAFs. Despite these strengths, this study has certain limitations. First, the findings rely primarily on a single ESCC cell line, which may limit the generalizability of the results. Second, all functional observations were made *in vitro*, and *in vivo* validation is required to confirm the physiological relevance of hypoxic migrasomes in tumor–stromal communication. In addition, the RNA-seq analysis was conducted under an exploratory framework without multiple testing correction, and thus, the transcriptomic findings should be interpreted with caution. The specific hypoxic migrasome cargo driving fibroblast activation also remains unidentified. Furthermore, our monoculture model does not fully reflect the complexity of the tumor stroma.

## Conclusion

In summary, our findings suggest that hypoxic migrasomes from ESCC cells may promote CAF-like activation in normal fibroblasts *in vitro*. Hypoxia altered the RNA cargo (mRNA, lncRNA, and circRNA) profiles of migrasomes, which were potentially involved in response to hypoxia, chromatin remodeling, ubiquitination, and metabolic processes. This research provides new insights into the multicellular ecosystem of ESCC and opens up a new field in the progression of ESCC study.

**Key Points**

- ESCC-derived migrasomes activate CAFs *via* hypoxia-altered RNA cargo.
- Hypoxia reshapes migrasomal RNA profiles, altering metabolic/epigenetic pathways.
- Hypoxic migrasomes enhance fibroblast migration/pro-inflammatory cytokine secretion.

## Supplementary Material

Fig_S1_elag002

Table_S1_elag002

Table_S2_elag002

Table_S3_elag002

Table_S4_elag002

Table_S5_elag002

MRC-5_Cell_STR_Identification_elag002

kyse-150_Cell_STR_Identification_elag002

## Data Availability

The datasets generated during the current study are available in the NCBI SRA repository under accession number PRJNA1301623.

## Notes

**Guan’en Qiao**, M.D., Ph.D., is a Professor and Chief Physician at The First Affiliated Hospital of Soochow University and Handan First Hospital. His research focuses on the molecular mechanisms of gastrointestinal cancers.

**Zhongping Wang**, M.D., is an Associate Professor at Handan First Hospital. She is engaged in clinical research with an interest in cancer-related systemic effects.

**Meng Wang**, B.S., is a Charge Nurse at Handan First Hospital. She participates in clinical research and patient care coordination for oncology studies.

**Le Feng**, B.S., is an Associate Professor of Nursing at Handan First Hospital. She contributes to clinical research protocols and nursing management in gastroenterology.

**Zixuan You**, M.S., is an Attending Physician at Handan First Hospital. She is involved in clinical research and the diagnosis of gastrointestinal tumors.

**Bing Meng**, M.S., is a Laboratory Technologist at Handan First Hospital. She specializes in experimental techniques and data analysis for biomedical research.

**Kui Dong**, M.S., is an Attending Physician at Handan First Hospital. His work integrates clinical practice with research on gastrointestinal tumor biology.

**Yanping Cao**, M.D., Ph.D., is a Chief Physician and heads the Central Laboratory at Handan First Hospital. She leads translational research on disease pathogenesis.

**Pan Li**, M.S., is an Attending Physician at Handan First Hospital. She participates in clinical studies aimed at advancing digestive cancer treatment.

**Junhai Wang**, M.S., is an Assistant Research Fellow at Handan First Hospital. He supports experimental investigations into disease mechanisms in the central laboratory.

**Xinqing Lu**, M.D., Ph.D., is a Professor at Handan First Hospital. His research is dedicated to translating discoveries in gastrointestinal oncology to clinical practice.

**Chunfang Xu**, M.D., Ph.D., is a Professor and Chief Physician at The First Affiliated Hospital of Soochow University. He is a leading researcher in gastrointestinal oncology, focusing on molecular carcinogenesis and novel therapies.
