# Supplementary figures and images for: Whole-transcriptome sequencing reveals hypoxic esophageal squamous cell carcinoma–derived migrasomes driving cancer-associated fibroblast activation
Source: Brief Funct Genomics. 2026 Jun 2;25:elag002. doi: 10.1093/bfgp/elag002 (PMC13229262; doi:10.1093/bfgp/elag002)

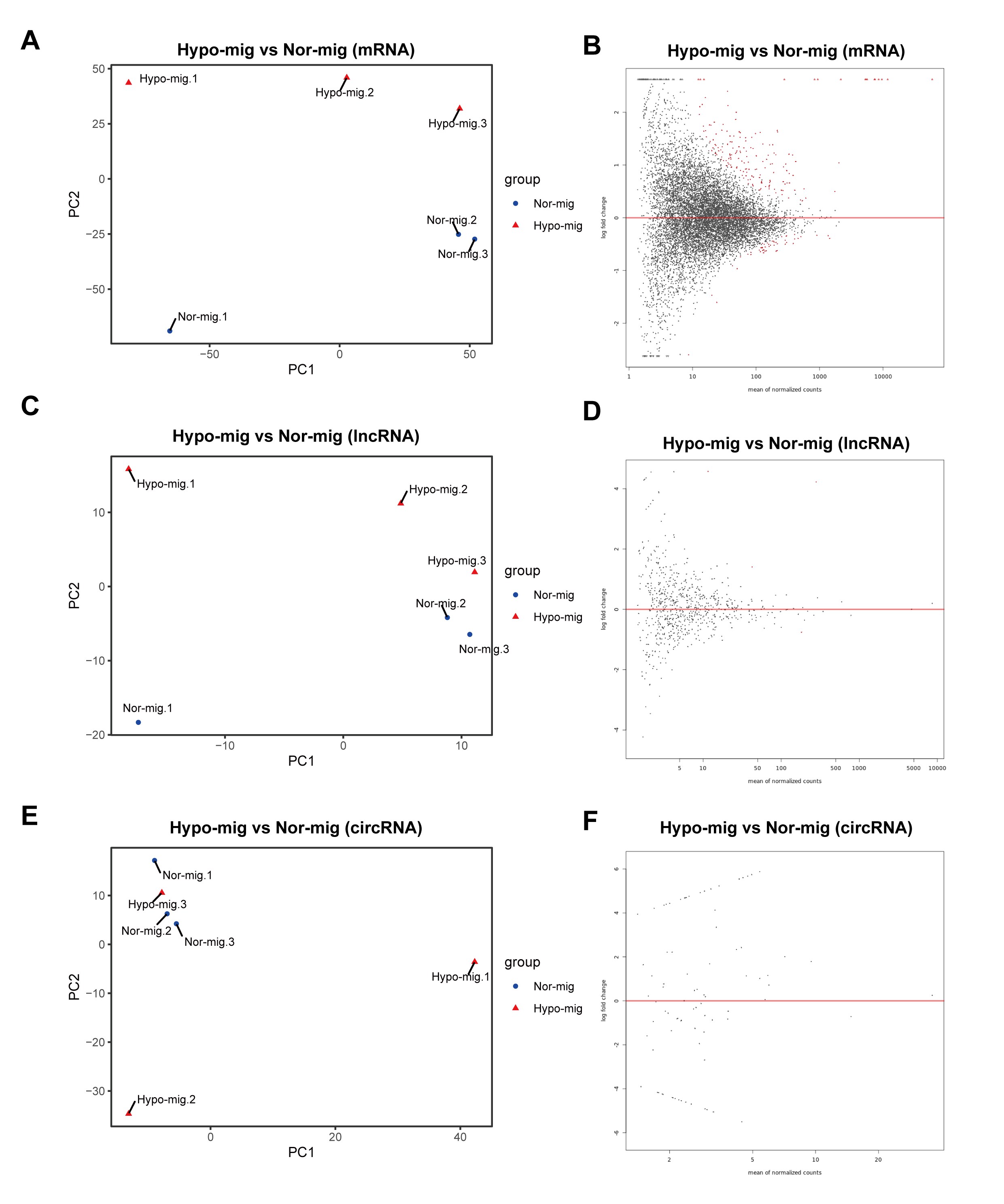

Supplement: Fig_S1_elag002 [file fig_s1_elag002.jpeg]
