# Supplementary material for: Whole-transcriptome sequencing reveals hypoxic esophageal squamous cell carcinoma–derived migrasomes driving cancer-associated fibroblast activation
Source: Brief Funct Genomics. 2026 Jun 2;25:elag002. doi: 10.1093/bfgp/elag002 (PMC13229262; doi:10.1093/bfgp/elag002)
Supplement: Table_S1_elag002 [file table_s1_elag002.docx]

**Table S1. Primer sequences**

| **Name** | **Sequences (5’ to 3’)** |
| --- | --- |
| Actin-F | AGCACAGAGCCTCGCCTTTG |
| Actin-R | CTTCTGACCCATGCCCACCA |
| FAP-F | CAAAGGCTGGAGCTAAGAATCC |
| FAP-R | ACTGCAAACATACTCGTTCATCA |
| PDGFRβ-F | GTGGTTGAGAGCGGCTACG |
| PDGFRβ-R | AGGGTGCGGTTGTCTTTGAA |
| ATP8-F | CACCTACCTCCCTCACCAAA |
| ATP8-R | GCAATGAATGAAGCGAACAG |
| ND4L-F | TCGCTCACACCTCATATCCTC |
| ND4L-R | GCTAAGAGGGAGTGGGTGTT |
| ND3-F | CCCTACCATGAGCCCTACAA |
| ND3-R | GGCCAGACTTAGGGCTAGGA |
| MTND1P23-F | TAACGCACTCTCCCCTGAAC |
| MTND1P23-R | GGGGAATGCTGGAGATTGTA |
| MTND2P28-F | CCGGACAATGAACCATAACC |
| MTND2P28-R | TCAGAAGTGAAAGGGGGCTA |
| UCA1-F | CGGGTAACTCTTACGGTGGA |
| UCA1-R | TAGGGTCTGGTCCATTGAGG |
| circRNA-ASXL1-F | TATAAACTGCCTGGCCGAAT |
| circRNA-ASXL1-R | TGCCTCTATGACCTGCAGAA |
| FAP-F | CAAAGGCTGGAGCTAAGAATCC |
| FAP-R | ACTGCAAACATACTCGTTCATCA |
| PDGFRβ-F | GTGGTTGAGAGCGGCTACG |
| PDGFRβ-R | AGGGTGCGGTTGTCTTTGAA |
