# Supplementary material for: Whole-transcriptome sequencing reveals hypoxic esophageal squamous cell carcinoma–derived migrasomes driving cancer-associated fibroblast activation
Source: Brief Funct Genomics. 2026 Jun 2;25:elag002. doi: 10.1093/bfgp/elag002 (PMC13229262; doi:10.1093/bfgp/elag002)
Supplement: Table_S2_elag002 [file table_s2_elag002.docx]

**Table S2. Sample sequencing depth and mapping statistics**

| **Sample Name** | **Total Reads** | **TotalReads**  **Before** | **TotalBase**  **Before** | **TotalReads**  **After** | **TotalBase**  **After** | **ReadsFilter**  **%** | **BaseFilter**  **%** | **GC%**  **Before** | **GC%**  **After** | **Mapping Ratio (%)** | | **Mapped**  **Base** |
| --- | --- | --- | --- | --- | --- | --- | --- | --- | --- | --- | --- | --- |
| Nor-mig-1 | 112164342 | 112164342 | 16824594641 | 110074542 | 16493383289 | 0.98136841 | 0.980313858 | 44 | 43.5 | 1.1 | 172345988 | |
| Nor-mig-2 | 80171368 | 80171368 | 12025664266 | 78795494 | 11806827108 | 0.982838337 | 0.981802489 | 43 | 43 | 1.2 | 127765732 | |
| Nor-mig-3 | 86827694 | 86827694 | 13024110839 | 83299160 | 12479222481 | 0.959361653 | 0.958163105 | 44.5 | 44 | 1.1 | 123243486 | |
| Hypo-mig-1 | 93167976 | 93167976 | 13975149628 | 91398824 | 13691492607 | 0.981011158 | 0.979702756 | 44 | 44 | 2.2 | 276671530 | |
| Hypo-mig-2 | 63589806 | 63589806 | 9538438756 | 57229222 | 8554991959 | 0.899974785 | 0.896896461 | 47 | 44 | 2.6 | 210328941 | |
| Hypo-mig-3 | 87412930 | 87412930 | 13111896027 | 82157260 | 12302030372 | 0.939875371 | 0.938234283 | 46.5 | 45 | 1.6 | 189486105 | |
