# Supplementary material for: Whole-transcriptome sequencing reveals hypoxic esophageal squamous cell carcinoma–derived migrasomes driving cancer-associated fibroblast activation
Source: Brief Funct Genomics. 2026 Jun 2;25:elag002. doi: 10.1093/bfgp/elag002 (PMC13229262; doi:10.1093/bfgp/elag002)
Supplement: Table_S3_elag002 [file table_s3_elag002.docx]

**Table S3. The top 10 differentially expressed mRNAs**

| **AccID** | **log2FC** | **Pvalue** | **FDR** | **Hypo-mig -1**  **(expression)** | **Hypo-mig -2**  **(expression)** | **Hypo-mig -3**  **(expression)** | **Nor-mig-1**  **(expression)** | **Nor-mig-2**  **(expression)** | **Nor-mig-3**  **(expression)** | **Style** |
| --- | --- | --- | --- | --- | --- | --- | --- | --- | --- | --- |
| ATP8 | 4.860351214 | 1.45E-29 | 8.41E-27 | 929 | 391 | 455 | 31 | 16 | 10 | up |
| ND4L | 4.123109741 | 1.32E-218 | 4.61E-215 | 5339 | 3923 | 3836 | 302 | 223 | 178 | up |
| ND3 | 4.057833085 | 1.53E-193 | 3.56E-190 | 2561 | 1572 | 1582 | 127 | 102 | 87 | up |
| ND1 | 4.021967334 | 1.21E-121 | 2.12E-118 | 20568 | 12035 | 12090 | 1296 | 703 | 597 | up |
| ND2 | 3.933453142 | 2.54E-28 | 1.18E-25 | 16381 | 7676 | 8237 | 1022 | 585 | 379 | up |
| ATP6 | 3.8856932 | 4.21E-23 | 1.73E-20 | 17414 | 8025 | 9054 | 1214 | 586 | 418 | up |
| COX1 | 3.774037327 | 0 | 0 | 148055 | 107913 | 99468 | 10386 | 7298 | 6556 | up |
| COX3 | 3.77372807 | 1.23E-119 | 1.71E-116 | 20047 | 12264 | 12580 | 1424 | 974 | 681 | up |
| ND6 | 3.675024834 | 2.37E-14 | 5.17E-12 | 2591 | 1100 | 1377 | 227 | 89 | 66 | up |
| ND4 | 3.502643215 | 8.70E-33 | 7.59E-30 | 32995 | 18147 | 19296 | 3028 | 1663 | 1205 | up |
