# Supplementary material for: Whole-transcriptome sequencing reveals hypoxic esophageal squamous cell carcinoma–derived migrasomes driving cancer-associated fibroblast activation
Source: Brief Funct Genomics. 2026 Jun 2;25:elag002. doi: 10.1093/bfgp/elag002 (PMC13229262; doi:10.1093/bfgp/elag002)
Supplement: Table_S4_elag002 [file table_s4_elag002.docx]

**Table S4. The top 10 differentially expressed lncRNAs**

| **AccID** | **log2FC** | **Pvalue** | **FDR** | **Hypo-mig -1**  **(expression)** | **Hypo-mig -2**  **(expression)** | **Hypo-mig -3**  **(expression)** | **Nor-mig-1**  **(expression)** | **Nor-mig-2**  **(expression)** | **Nor-mig-3**  **(expression)** | **Style** |
| --- | --- | --- | --- | --- | --- | --- | --- | --- | --- | --- |
| MTND2P28 | 4.223409041 | 1.07E-58 | 7.67E-56 | 786 | 379 | 506 | 44 | 29 | 17 | up |
| MTND1P23 | 4.572743559 | 4.89E-06 | 0.001756383 | 34 | 24 | 13 | 3 | 0 | 0 | up |
| UCA1 | 1.410036833 | 2.43E-05 | 0.005816677 | 89 | 56 | 49 | 33 | 21 | 19 | up |
| MTND4P12 | 4.561873425 | 0.001531692 | 0.219951021 | 12 | 9 | 5 | 1 | 0 | 0 | up |
| LOC100131294 | 1.50718646 | 0.002937786 | 0.35155502 | 53 | 29 | 15 | 13 | 6 | 14 | up |
| LOC100288181 | 1.437462272 | 0.007371922 | 0.708129839 | 29 | 26 | 14 | 8 | 7 | 10 | up |
| SCARNA6 | 4.556223802 | 0.008876279 | 0.708129839 | 1 | 3 | 8 | 0 | 0 | 0 | up |
| LOC101927861 | 3.865808131 | 0.013321088 | 0.782311513 | 7 | 6 | 3 | 0 | 0 | 1 | up |
| LOC100130388 | 4.310852606 | 0.013897718 | 0.782311513 | 5 | 2 | 4 | 0 | 0 | 0 | up |
| TAF9P3 | 3.898402321 | 0.014164415 | 0.782311513 | 11 | 4 | 2 | 1 | 0 | 0 | up |
