# Supplementary material for: Whole-transcriptome sequencing reveals hypoxic esophageal squamous cell carcinoma–derived migrasomes driving cancer-associated fibroblast activation
Source: Brief Funct Genomics. 2026 Jun 2;25:elag002. doi: 10.1093/bfgp/elag002 (PMC13229262; doi:10.1093/bfgp/elag002)
Supplement: Table_S5_elag002 [file table_s5_elag002.docx]

**Table S5. Differentially expressed circRNAs**

| **AccID** | **log2FC** | **Pvalue** | **FDR** | **Hypo-mig -1**  **(expression)** | **Hypo-mig -2**  **(expression)** | **Hypo-mig -3**  **(expression)** | **Nor-mig-1**  **(expression)** | **Nor-mig-2**  **(expression)** | **Nor-mig-3**  **(expression)** | **Style** |
| --- | --- | --- | --- | --- | --- | --- | --- | --- | --- | --- |
| chr20_32369123_32366384_+2739-ASXL1 | 5.544356 | 0.032001 | 0.461238 | 8 | 8 | 8 | 0 | 0 | 0 | up |
