# Supplementary material for: Whole-transcriptome sequencing reveals hypoxic esophageal squamous cell carcinoma–derived migrasomes driving cancer-associated fibroblast activation
Source: Brief Funct Genomics. 2026 Jun 2;25:elag002. doi: 10.1093/bfgp/elag002 (PMC13229262; doi:10.1093/bfgp/elag002)
Supplement: kyse-150_Cell_STR_Identification_elag002 [file kyse-150_cell_str_identification_elag002.doc]

1. **STR基因型检测报告**

**样本名称：KYSE-150**

**检测方法：**用Axygen的基因组抽提试剂盒提取DNA，采用20- STR扩增方案扩增，在ABI 3730XL型遗传分析仪上对STR位点和性别基因Amelogenin进行检测。

**检测结果：**该株细胞DNA分型在细胞系检索中找到**完全匹配**的细胞系，DSMZ数据库显示细胞名为**KYSE-150**，细胞号对应**375**。本次检测在该细胞系中**没有发现多等位基因**。细胞匹配值：1.0

**STR数据库比对分析：**待测细胞的STR位点和Amelogenin位点的基因分型结果与收录于ExPASY,ATCC, DSMZ, JCRB 和 RIKEN数据库的2455个细胞系STR数据进行比对，如果待检测细胞未收录于以上细胞库或这是自行建立的新细胞系将无法进行比对，用户需根据细胞分型结果自行与其他数据库进行比对。

**分型结果：**

| Loci | 送检细胞STR信息 | | | 细胞库细胞STR信息 | | |
| --- | --- | --- | --- | --- | --- | --- |
|  | 送检细胞名：kyse-150 | | | 细胞库细胞名：KYSE-150 | | |
|  | Allele1 | Allele2 | Allele3 | Allele1 | Allele2 | Allele3 |
| D5S818 | 12 | 13 |  | 12 | 13 |  |
| D13S317 | 8 | 11 |  | 8 | 11 |  |
| D7S820 | 10 | 11 |  | 10 | 11 |  |
| D16S539 | 9 | 11 |  | 9 | 11 |  |
| VWA | 16 | 17 |  | 16 | 17 |  |
| TH01 | 7 | 9 |  | 7 | 9 |  |
| AMEL | X | X |  | X | X |  |
| TPOX | 8 | 8 |  | 8 | 8 |  |
| CSF1PO | 12 | 13 |  | 12 | 13 |  |
| D12S391 | 19 | 22 |  |  |  |  |
| FGA | 21 | 24 |  |  |  |  |
| D2S1338 | 25 | 25 |  |  |  |  |
| D21S11 | 30 | 31 |  |  |  |  |
| D18S51 | 14 | 14 |  |  |  |  |
| D8S1179 | 10 | 15 |  |  |  |  |
| D3S1358 | 15 | 16 |  |  |  |  |
| D6S1043 | 18 | 20 |  |  |  |  |
| PENTAE | 12 | 18 |  |  |  |  |
| D19S433 | 15 | 15 |  |  |  |  |
| PENTAD | 10 | 10 |  |  |  |  |
| D1S1656 | 15 | 17.3 |  |  |  |  |

**分型图谱：**


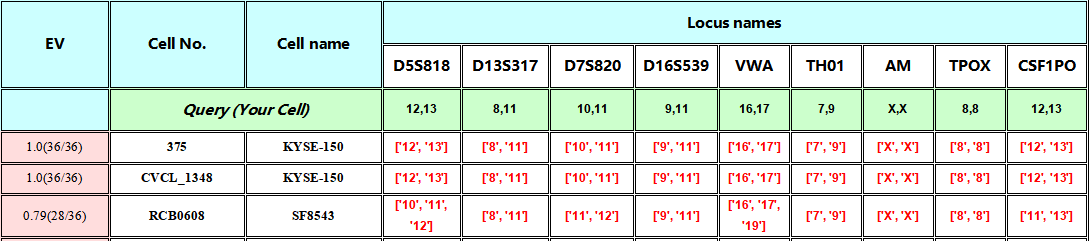

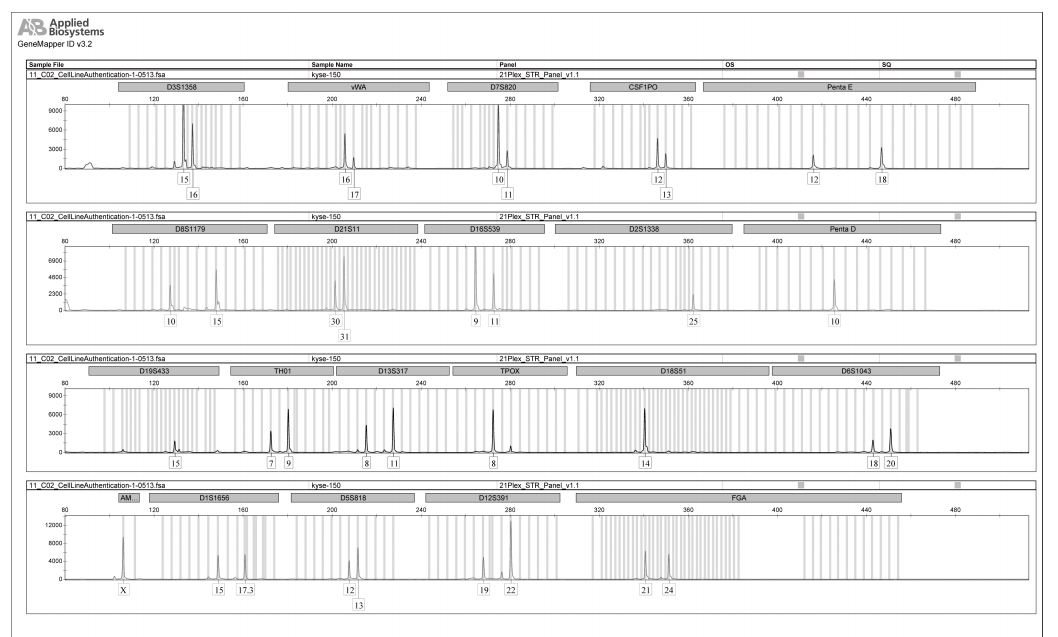

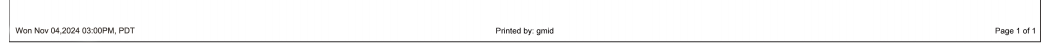


**备注：**

1. 根据国际细胞鉴定委员会（ICLAC)制定的细胞STR鉴定标准，匹配度≥80%即可认为该细胞系正确，匹配度＜80%则说明该细胞系的来源需要被怀疑。
2. 图谱有效峰位真实的PCR条带，小峰和非特异性条带在计算中忽略不计。
3. 国家实验细胞资源共享平台，数据库入口http://www.cellresource.cn /。
